# Supplementary material for: RGFP966 inhibits activation of AIM2 inflammasomes to promote mitophagy to relieve acute gouty arthritis
Source: PLoS One. 2026 May 14;21(5):e0348274. doi: 10.1371/journal.pone.0348274 (PMC13175381; doi:10.1371/journal.pone.0348274)
Supplement: S1 File — (PDF) [file pone.0348274.s001.pdf]

Figure 5.

1. AIM2

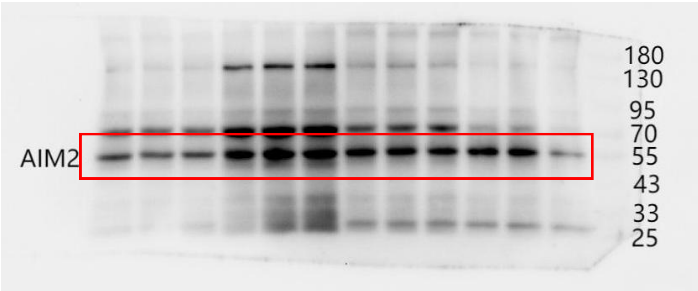

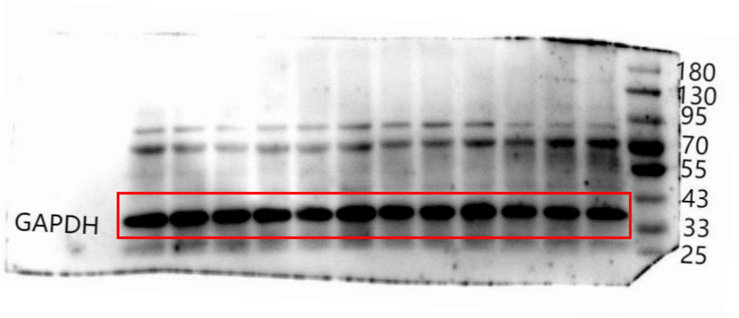

2. ASC

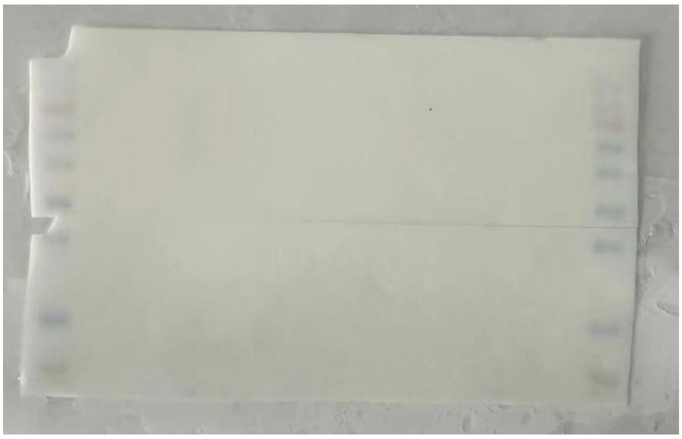

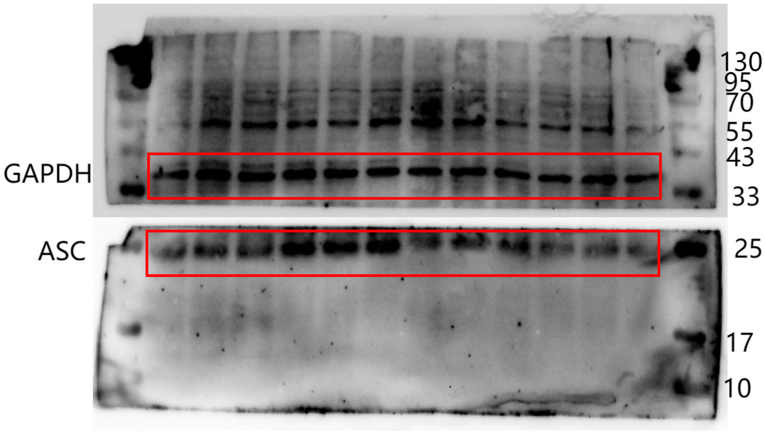

uncropped images

3. Pro-caspase-1

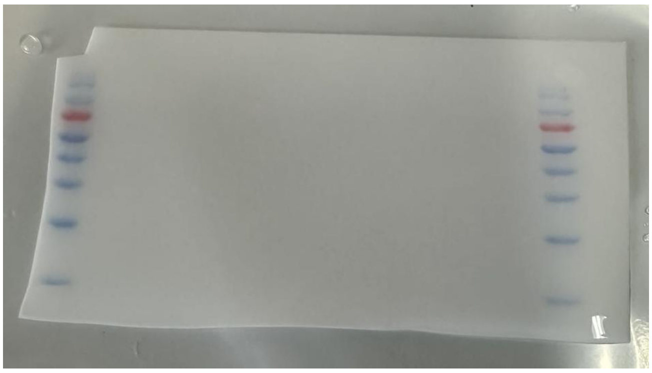

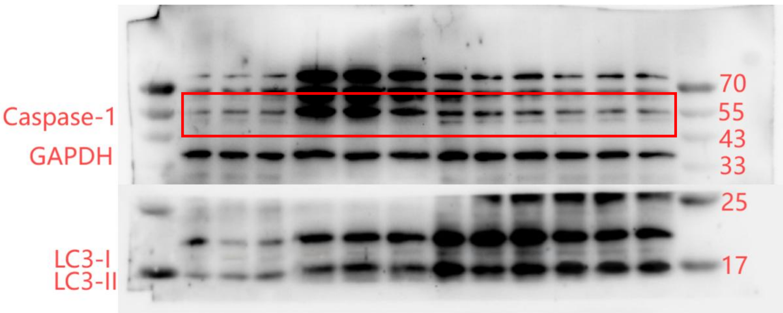

uncropped images

4. Cleaved-caspase-1

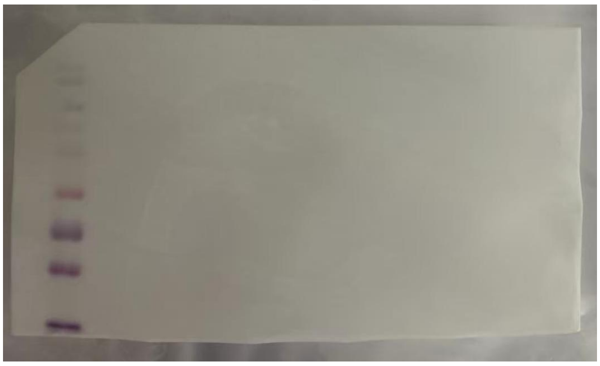

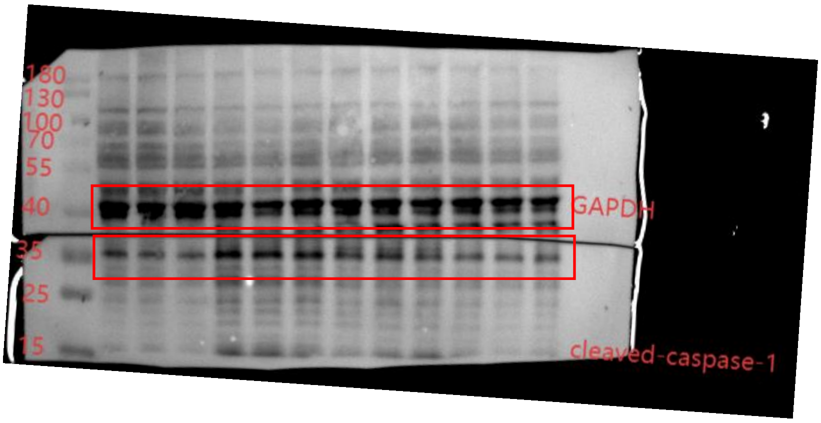

uncropped images

5. Pro-IL-1β

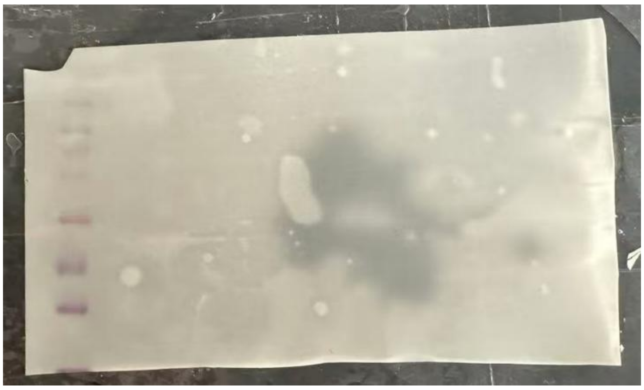

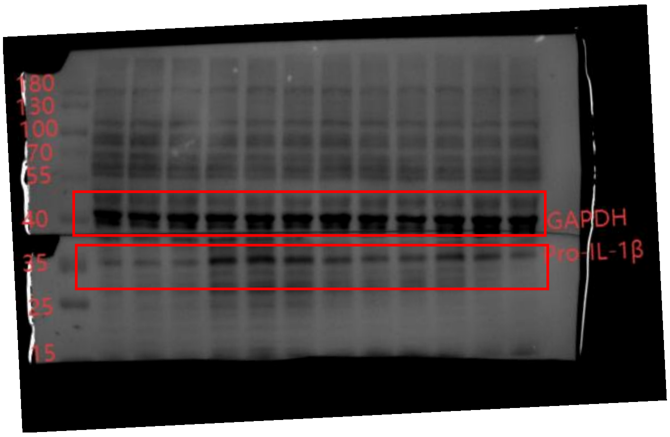

uncropped images

6. Cleaved-IL-1β

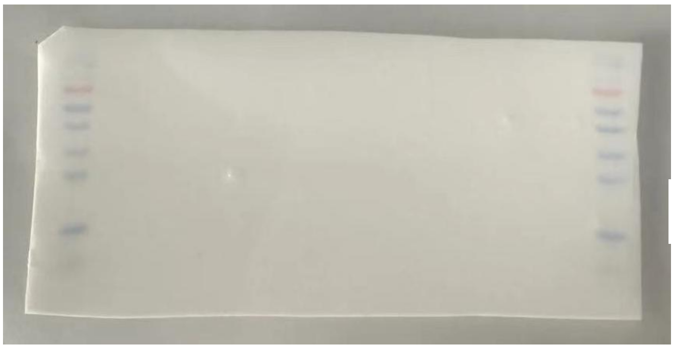

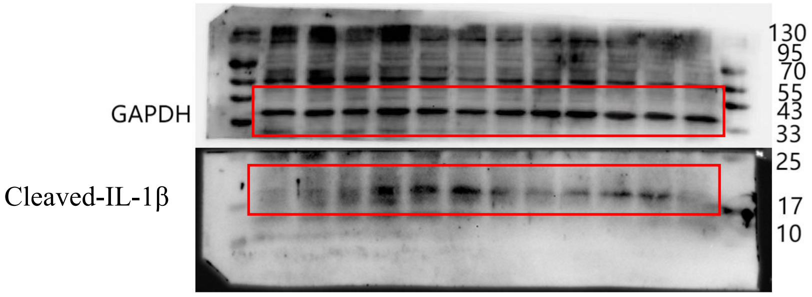

uncropped images

Figure 7.

1.Pink1

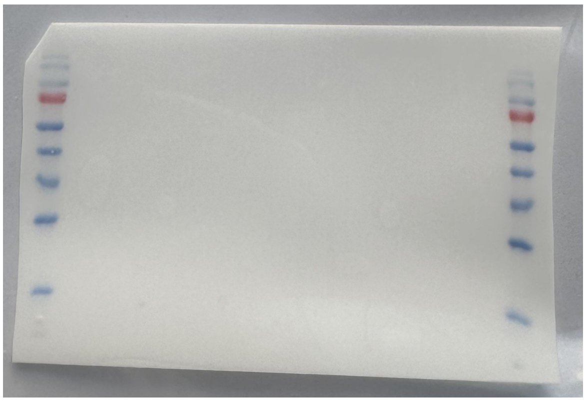

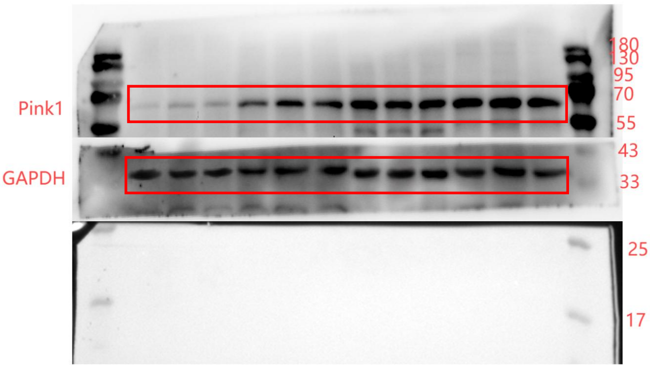

uncropped images

2. Parkin

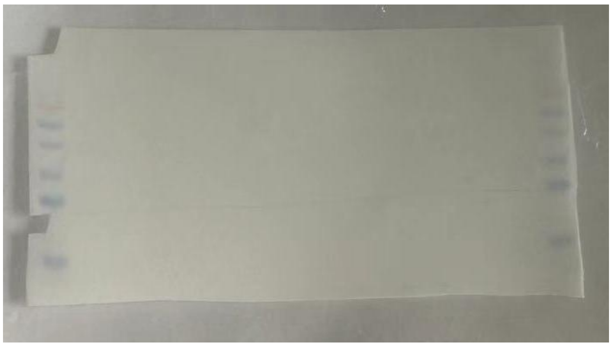

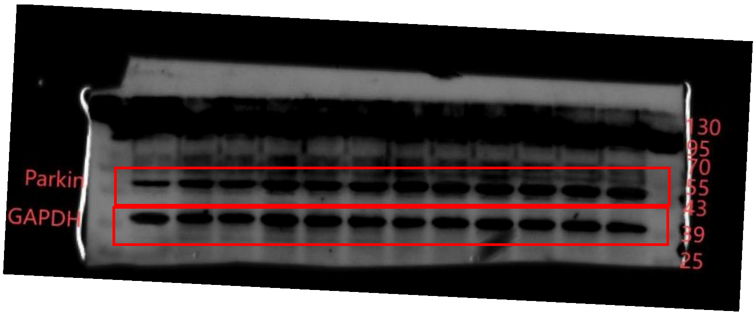

uncropped images

3. LC3

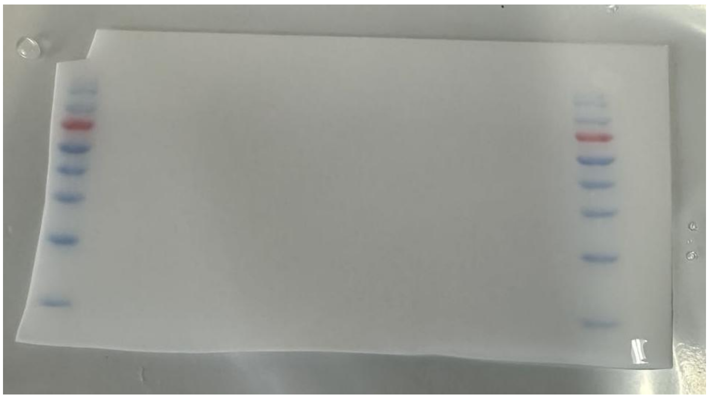

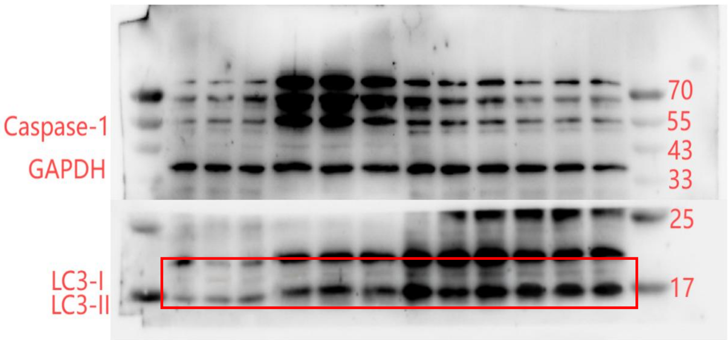

uncropped images
