# Supplementary material for: RGFP966 inhibits activation of AIM2 inflammasomes to promote mitophagy to relieve acute gouty arthritis
Source: PLoS One. 2026 May 14;21(5):e0348274. doi: 10.1371/journal.pone.0348274 (PMC13175381; doi:10.1371/journal.pone.0348274)
Supplement: Attachment — Submitted filename: review.docx [file pone.0348274.s003.docx]

The concept of the study is good and the experimental design is strong; however, the manuscript would benefit from improved organization, as well as corrections to English and grammar.

1. **Introduction:** While AIM2 activation is described, AGA is primarily associated with NLRP3 inflammasome activation. The manuscript does not justify the emphasis on AIM2 over NLRP3. Additionally, the final paragraph should clearly state the study objective and hypothesis; methodological details are better suited for the Methods section.
2. **Methods (Line 95):** The group descriptions are unclear. Please specify whether Group 3 represents the RGFP966-only treatment group or RGFP966 combined with MSU.
3. **Line 134:** The word “cuted” is incorrect and should be replaced with “cut.”
4. **Line 135 (mitochondrial extraction method):** Providing the centrifugation speed as an RCF value would improve reproducibility.

Addressing these points will improve the clarity and readability of the manuscript
