# Supplementary material for: RGFP966 inhibits activation of AIM2 inflammasomes to promote mitophagy to relieve acute gouty arthritis
Source: PLoS One. 2026 May 14;21(5):e0348274. doi: 10.1371/journal.pone.0348274 (PMC13175381; doi:10.1371/journal.pone.0348274)
Supplement: Attachment — Submitted filename: Response to Reviewer.docx [file pone.0348274.s005.docx]

**Response to Reviewer’s**

**Manuscript Number: PONE-D-25-67001**

**Title: RGFP966 inhibits activation of AIM2 inflammasomes to promote mitophagy to relieve** **acute gouty arthritis**

Dear editor and reviewer,

Thank for your professional review on my article. As you are concerned, there are some problems that need to be addressed. According to your nice suggestions, we have made extensive corrections to our previous draft, and the changes are highlighted in the manucript. Our point-to-point responses are as follows.

Reviewer#1:

The authors should clarify their findings. They should shorten their discussion, and their results. They should compare their findings with those of other authors within the discussion. The manuscript should have some figures depicting the results.

Response: Thanks for your positive comments. Based on your comments, we have shorten discussion and results. We have added some figures depicting the results in the Results section in the revised research. Some findings have listed compared with our findings in the Discussion section.

Reviewer#2:

We sincerely thank the reviewer for careful reading. However, this reviewer's comments are unrelated to my manuscript.

Reviewer#3:

1. Introduction: While AIM2 activation is described, AGA is primarily associated with NLRP3 inflammasome activation. The manuscript does not justify the emphasis on AIM2 over NLRP3. Additionally, the final paragraph should clearly state the study objective and hypothesis; methodological details are better suited for the Methods section.

Response: We would like to express our sincere gratitude for editor’ comments and valuable feedback. The revised content of the paper is as follows: Inflammasomes, composed of pattern recognition receptors (PRRs), apoptosis-associated speck-like protein containing an apoptosis-associated speck-like protein (ASC), and inactive pro-caspase-1, are multiprotein complexes found in the cytoplasm [6]. They can only function after they are activated. The PRRs of inflammasomes mainly include members of the nucleotide-binding oligomerization domain-like receptor (NLR) family and the absent in melanoma 2 (AIM2)-like receptor (ALR) family [7]. (Page 3)

References：

6. Lu A, Magupalli VG, Ruan J, Yin Q, Atianand MK, Vos MR, et al. Unified polymerization mechanism for the assembly of ASC-dependent inflammasomes. Cell.2014;156:1193–206. doi: 10.1016/j.cell.2014.02.008

7. Chu J, Tian J, Li P, Fu D, Guo L, Sun R. The impact of AIM2 inflammasome-induced pyroptosis on acute gouty arthritis and asymptomatic hyperuricemia patients. Front Immunol. 2024;15:1386939. doi: 10.3389/fimmu.2024.1386939.

In order to reveal the role and mechanism of RGFP966-mediated AIM2 inflammasomes in regulation of Pink1/Parkin signaling pathway, this study established an AGA rat model and used colchicine as the positive control. We investigated the effects of RGFP966, a potential AIM2 inhibitor, on inflammatory and mitophagy-related markers in rat synovial tissues, thereby providing new insights into potential therapeutic targets for gout prevention and treatment. (Page 5)

2.The group descriptions are unclear. Please specify whether Group 3 represents the RGFP966-only treatment group or RGFP966 combined with MSU.

Response: Considering the editors’s suggestion, we have corrected into RGFP966+MSU and Colchicine+MSU in the Experiment design section on page 5.

3. The wordcuted” is incorrect and should be replaced with “cut.”

Response: We sincerely thank the reviewer for careful reading. As suggested by the reviewer, we have corrected into “cut”.

4. Providing the centrifugation speed as an RCF value would improve reproducibility.

Response: We sincerely thank the reviewer for careful reading. As suggested by the reviewer, we have provided the centrifugation speed on page 7.
