# Supplementary material for: RGFP966 inhibits activation of AIM2 inflammasomes to promote mitophagy to relieve acute gouty arthritis
Source: PLoS One. 2026 May 14;21(5):e0348274. doi: 10.1371/journal.pone.0348274 (PMC13175381; doi:10.1371/journal.pone.0348274)
Supplement: Attachment — Submitted filename: Response to Reviewers.docx [file pone.0348274.s006.docx]

**Response to Reviewers**

**Manuscript Number: PONE-D-25-67001**

**Title: RGFP966 inhibits activation of AIM2 inflammasomes to promote mitophagy to relieve** **acute gouty arthritis**

Dear editor and reviewer,

Thank for your professional review on my article. As you are concerned, there are some problems that need to be addressed. According to your nice suggestions, we have made extensive corrections to our previous draft, and the changes are highlighted in the manucript. Our point-to-point responses are as follows.

Reviewer#1:

Reviewer #1: The authors should improve the use of the English language. The use of the English language should be improved throuhout the manuscript including the abstract, the introduction, the methods section, the results section and of course the discussion and the conclusions section. If the use of the English language is improved it will be easier for the readers to comprehend the meaning of the paper and to understand the aim and significance of the experiments which are described in sufficient detail. Improvement of the use of the English language will make the paper understandable to a large audience all over the world.

Response: Thanks for your positive comments. Based on your comments, we tried our best to improve the manuscript and polish the language. These changes will not influence the content and framework of the paper. And here we did not list the changes but marked in red in the revised paper. We appreciate for Editors/Reviewers’ warm work earnestly and hope that the correction will meet with approval.
